# “Nobody Will Believe Me”: Lack of Reporting Violence by Disabled Women

**DOI:** 10.1177/10778012251384622

**Published:** 2025-10-07

**Authors:** Eliona Gjecaj, Rannveig Traustadóttir, James Gordon Rice

**Affiliations:** 1 Centre for Disability Studies, School of Social Sciences, 63541 University of Iceland, Reykjavík, Iceland

**Keywords:** disabled women, violence, lack of reporting, access to justice, Iceland

## Abstract

A growing body of research shows the high risk, frequency, forms, and impact of violence against disabled women throughout their lives. Limited research exists regarding access to justice for disabled women, in particular with reporting and prosecution. Using an interdisciplinary human rights approach, the paper draws on interviews with disabled women about their experiences of gender-based violence and access to justice. Findings describe and discuss their lived experiences of violence, highlighting the lack of reporting the violence and the multiple and complex barriers to doing so. The article concludes with recommendations that may help to overcome these barriers.

## Introduction

International research demonstrates that disabled women are at a higher risk of experiencing violence than other women. They also experience violence for longer periods of time, and a wider range of forms, than non-disabled women (Barrett et al., 2009; Emerson & Llewellyn, 2023; Hughes et al., 2012; Krnjacki et al., 2016; Manjoo, 2012; McConnell & Phelan, 2022; Snæfríðar-Gunnarsdóttir et al., 2023). Most of the acts of violence against disabled women occur at home, and partners and/or caregivers are most likely to be the perpetrators (Cotter, 2018; Hughes et al., 2012; Plummer & Findley, 2012). Research also indicates that marginalization and isolation exacerbate the vulnerability of disabled women to abuse and that the effects of violence may be magnified when women occupy multiple marginalized subject positions (Björnsdóttir et al., 2017; Goodman et al., 2009; Meer & Combrinck, 2015), resulting in most women internalizing the oppression and experiencing feelings of powerlessness and self-blame (Matheson et al., 2015; Traustadóttir & Snæfríðar-Gunnarsdóttir, 2014; Walter-Brice et al., 2012). Limited research has focused on access to justice for disabled women who have been subjected to gender-based violence, in particular with regard to reporting and prosecution, both internationally and in Iceland, where the research presented in this paper was conducted. This interdisciplinary research employs a human rights approach in combining disability studies, gender studies, and disability law and policy, together with qualitative methods, to gain a comprehensive view of this topic. This study seeks to advance the understanding of violence against disabled women through documenting their lived experiences and analyzing the barriers they encountered in reporting the violence.

*Reporting* in this study refers to any act of reporting the violence to the police or other agencies (including victim and/or disability support services). Drawing attention to the under-reporting of violence by disabled people, Davis (2011) states that while there are disproportionately high rates of violence against disabled people, the rates at which such violence is reported to the police are disproportionately low. Flynn and Lawson (2013) observe that barriers to accessing justice encountered by disabled people tend to vary based on intersectional identities and the types of impairments. The available data show that disabled women face significant challenges in accessing justice and protection (Benedet & Grant, 2014; Budu-Ainooson et al., 2020; McCulloch et al., 2021; McGowan & Elliott, 2019; Rugoho et al., 2022; Wulandari, 2018). One particular barrier relevant to disabled women is the lack of the support and socialization needed in order to recognize certain types of behavior (especially emotional or verbal) as abusive and therefore as a wrong worthy of reporting (Powers & Oschwald, 2004). Gender issues (e.g., dealing with male police) and physical and communication barriers have also been shown as effecting whether disabled women report violence or not (Dowse et al., 2016; Hester & Lilley, 2018; McCulloch et al., 2021; Rugoho et al., 2022). Other barriers which discourage the pursuit of legal redress include dismissive attitudes, harmful stereotypes, discrimination, problematic reporting procedures (CRPD Committee, 2016, p. 52; Rugoho et al., 2022; Wulandari, 2018), and intimidation by criminals and law enforcement (Wulandari, 2018). However, even when women disclose violence, research suggest that responses have the potential to be re-victimizing through words and actions, such as reactions that are dismissive or minimize their experiences, making disabled women feel worthless and powerless (Walter-Brice et al., 2012). The perception of disabled women as lacking credibility—by both law enforcement and family members—significantly diminishes their likelihood of reporting gender-based violence or engaging in follow-up processes (Rugoho et al., 2022). Even if the women's concerns are taken seriously, the services provided are not always appropriate to their needs and can fail to deliver the desired justice (Douglas & Harpur, 2016; McCulloch et al., 2021; Walter-Brice et al., 2012).

This literature, however, focusses primarily on violence prevention, or general support in the context of violence, while containing passing references to barriers to justice faced by disabled women. This paper offers a unique contribution through its detailed analysis of the barriers to justice faced by the disabled women who participated in the study—barriers that ultimately prevented them from reporting the violence and thus from initiating the process of seeking justice. The paper begins by outlining the interdisciplinary human rights approach to the study. It continues with a description of the research methodology and participants. The findings describe and discuss the lived experience of violence of disabled women and provide an analysis of the barriers to reporting. The paper concludes by discussing the meaning of justice in relation to these barriers and suggestions on how to remove them.

## Interdisciplinary Human Rights Approach

This research employs an interdisciplinary human rights approach in combining disability studies, gender studies and disability law and policy with the aim to gain a comprehensive and holistic view of access to justice for disabled women who have been subjected to gender-based violence. As noted by O'Mahony and Quinn (2017), the human rights approach is both driven and supported by the UN Convention on the Rights of Persons with Disabilities (CRPD, 2006), which signaled a “new era for people with disabilities” (Lawson, 2007, p. 619), recognizing disabled people and explicitly reiterating their standing as rights holders (Degener, 2016; Kanter, 2014). The Convention encapsulates a human rights perspective on disability and includes a clear articulation of the “different layers of identity” (Degener, 2016, p. 10), recognizing the aggravated forms of discrimination often faced by disabled persons on the basis of the combination of disability with other factors (Degener, 2017). Hence, it recognizes the multiple marginalization resulting from the intersection of disability and gender faced by disabled women, which is specifically outlined in Article 6 of the CRPD on Women and Girls with Disabilities.

Disability studies explores the lives of disabled people in social, cultural and political contexts and emphasizes the lived experiences of disabled individuals (Goodley, 2011) providing an important perspective for this research. Of particular importance is the relational approach focusing on how disability is created by societal barriers which contribute to social exclusion, powerlessness and marginalization of disabled individuals (Shakespeare, 2013). Gender studies have a long history of interdisciplinary research (Collins, 2009) addressing the multiple and intersecting forms of discrimination and power relations. This approach has informed the analysis of how gender, disability, and possibly other factors such as race, class and religion, expose disabled women to multiple systems of oppression (Barrett et al., 2009). Being both disabled and a woman causes a power imbalanced social status in most cultures (Hague et al., 2011). Hence, the intersection of gender and disability equate to double jeopardy, leaving disabled women more disempowered and vulnerable to violence than non-disabled women (Goodley & Runswick-Cole, 2011; Humphrey, 2016; Shah et al., 2016).

Violence is a concept that takes many forms and has multiple definitions. However, the way in which violence is understood, named, and categorized is connected to the prevailing ableist systems and ideologies (Mueller et al., 2019). Thus, understandings of violence are socially constructed, reflect power relations (Hollomotz, 2013), and often fail to grasp the different manifestations of violence that disabled women experience (Snæfríðar-Gunnarsdóttir et al., 2023). Therefore, employing an intersectional approach brings about a more nuanced understanding and reveals the complex and intersecting nature of violence, and helps to understand the barriers faced by disabled women regarding detecting, reporting and pursuing legal action against such violence.

## Methods

This paper is a part of the first author's doctoral research (Gjecaj et al., 2023, 2024), which explored the lived experience of disabled women as well as those supporting them through detection, reporting, investigation and prosecuting violence in Iceland. A qualitative research approach was employed to gather data on the lived experience of disabled women and those who assisted them in accessing the justice system (Taylor et al., 2016). Following the ethical review by the University of Iceland's Scientific Ethics Committee in April 2020, data collection started in August 2020 and ended in October 2022. Three qualitative methods have been used to gather data: 36 semi-structured interviews, document analysis (published life stories, court documents, national laws, policies, and international human rights treaties), and field observation (including court hearings and visits to victim support services). Grounded theory approach guided the data collection as well as a method for the ongoing data analysis alongside the data collection (Charmaz, 2014; Padgett, 2017).

This paper is informed by all of the data collected but the findings presented here are primarily based on 16 interviews with self-identified disabled women with a range of different impairments, ages, gender identities, ethnic backgrounds, education, and socio-economic status. Additionally, two life stories by disabled women were selected and analyzed as part of the study. Participants were identified through formal and informal networks and recruited using purposive sampling (Creswell, 2007) with an emphasis on creating a diverse group of participants to capture a broad range of experiences. A snowball methodology was also used as participants were asked to identify other possible participants. The women were between 18 and 68 years old. Their impairments were intellectual, psychological, physical and visual, autism and chronic illness. Two participants had multiple impairments. Participants lived in the capital area and the north region of Iceland. Four of the women were married or lived with a partner, three women were widowed or divorced, and five had children. Half, or eight, of the women were working or studying but the other half did not work. Three participants identified as non-binary. Most of the women were from Iceland apart from two who were of foreign origin.

Interviews were conducted in-person by the first author between August 2020 and October 2022. The interviews were arranged according to the preference of the participant and included settings such as the participants’ homes and an office at the university. All participants received an information sheet along with a consent form. Both were in Icelandic and English, and if requested in easy-to-read format. The information sheet detailed the aims and objectives of the study, voluntary participation, procedures for anonymity and confidentiality and the right to withdraw from the study, data storage and use of information. Consent forms were signed at the beginning of the interview or signed electronically and returned to the researcher by email. An interview guide developed by the research team (PhD committee) was used in the study. No definition of violence was provided but instead the women were invited to speak about what they had experienced and considered as violence. Questions were open-ended and exploratory in nature, investigating the experience of violence and of reporting it, or not, by the women. Given the sensitive nature of the topic, at the end of each interview all participants were offered consultation with a professional of their choice, free of charge, enabling them to work through potential difficult feelings or memories evoked by the interview. Each interview lasted approximately one to two hours, and were audio recorded with consent, and later transcribed verbatim. All names included in the findings are pseudonyms.

## Findings

### Experiences of Violence

The disabled women had experienced diverse types and forms of violence which differed according to age and over the life course. The timeframe of these experiences ranges from 1980 to the time of the interviews. Manifestations of violence varied from sexual, emotional, financial and physical violence to disability discrimination, microaggression, and prejudice. Several participants had experienced multiple types of violence throughout their life. Linda, for example, described violence as being “…a recurrent theme in my life. All kinds of violence”. In short, the women experienced a great number of forms of violence at the hands of people from most walks of life and in a range of social contexts.

Looking back on their lives, many of the women recalled experiences of violence during childhood. The most common form of violence was bullying, sexual violence, and combined types of violence. The women who had experienced bullying in primary school described the negative effect bullying and teasing had on them and their self-esteem, leaving them feeling lonely, isolated and having no friends. Some of the women had experienced violence in their childhood homes, including sexual abuse by family members. Another common manifestation of abuse was emotional violence and prejudice. In this regard the women talked of humiliation, prejudice, and overstepping of their boundaries as a common form of such violence. Perpetrators that subjected the women to violence in their childhood and teenage years included partners, friends, family members, school children, and teachers.

As young adults, the women commonly experienced intimate partner violence and various forms of sexual, emotional and physical violence. In these cases, the relationships had been characterized by an unequal power balance and control by the partner. The violence was also committed by former partners; for example, one of the women described the complicated relationship she had with her former boyfriend who, among other things, pressured her to do things she did not want to do including sexual acts with him or other men. One of the women was raped by her friend, and another was raped by two different strangers at separate times and a third time by a teacher at a continuing educational center.

As adults, in addition to partner violence, the women also described experiences of sexual violence perpetrated by staff of disability services, doctors, male colleagues, and male strangers. The scope of the violence included touching and fondling to sexual assaults, including rape. In all cases, the women highlighted the imbalance of power between them as disabled women and non-disabled perpetrators. Many participants also described excessive interference and forceful control of their lives by family members and staff within the disability services even though they were adults. Some participants, for example, described receiving inadequate support or experienced negative attitudes from staff. One of the women told of an abortion she had been forced to undergo by family members who also tried to coerce her to be sterilized. The interference and control by family members led to their relationships becoming estranged, negatively affecting the women's self-image and self-esteem, and left them in fear of further scolding, overprotection and control of their lives.

Furthermore, the women spoke about being subjected to disability prejudice and discrimination. Emotional violence was also highlighted as being profoundly related to prejudices against disabled people and intertwined with stereotypes and preconceptions regarding disabled women. Many of the participants had experienced this in a number of ways including people making fun of them, teasing, touching and staring at them. In some cases, the prejudice also led to other types of violence such as physical and sexual harassment. Hulda described how a fellow female student came over to her during a university trip and started feeling her arm, moving her hands, asking “What is wrong with you?” Overall, it can be observed that there is a relationship between the marginalization, powerlessness and subordinate status of disabled women and the violence to which they are subjected.

### Barriers to Reporting Violence

The most striking finding of this research was the lack of reporting these forms of violence. Only three of the 18 women made such reports. As a result, an in-depth analysis of the barriers to reporting became the major focus of our analysis revealing a range of complex and multilayered barriers discussed below. We have organized them into three broad categories: (1) personal (barriers from within); (2) interpersonal (barriers in relation to others); and (3) cultural and structural barriers. Each of these categories contain subthemes. Some of the categories and subthemes overlap as most of the women had multiple reasons for not reporting their perpetrators.

#### Personal Barriers

Many of the women described individual-level barriers to reporting. Subthemes included: unclear or partial understanding of violence; lack of preventative and timely knowledge about sexual violence; naming the assault; young age; and lack of knowledge on where to go for support or report. These subthemes overlap as discussed below.

#### Unclear or Partial Understanding of Violence

For many of the women the reason for not reporting was an unclear or partial understanding of what violence is. In particular, this became apparent in interviews with women who at the outset declared not having experienced violence. However, as the discussion progressed, they described experiences that were clearly acts of violence. Inga, for example, stated that she does not know what she would call violence and added that she has not experienced violence. However, as the interview continued, she described instances of experienced violence at a mental health institution. Jessie spoke about it being hard for them to recognize violence and that their misunderstanding of violence was due to their upbringing and their boundaries not being respected throughout their lives. Some of the women even stated that they had not experienced violence but rather “only prejudice”. Hulda declared that she has not experienced violence: “I have not been hit or anything, not that kind of violence”, but answered positively to having experienced prejudice: “No, no physical violence, but like I’ve had some fordómar (prejudice)”. Like the above women, Soley declared she had not experienced any type of violence or prejudice but rather what she described as violations of her rights: “I have not experienced it, except for in this way, like I have to try and get my rights you know, and maybe they violate my rights here and there you know, that's the only thing that has happened to me”. When asked for examples, she described instances of disability-based discrimination and psychological pressure.

#### Lack of Preventative and Timely Knowledge on Sexual Violence

Many of the women who experienced sexual violence highlighted the lack of preventative and timely knowledge on sexual violence before and after they experienced it. Hanna, who was subjected to three rapes by strange men when she was a young adult, recalls her lack, at the time, of knowledge about rape and the lack of information regarding the dangers of going into stranger's cars and the risks of sexual abuse associated with these behaviors: “I would have never have done this today but at the time you know I kind of didn’t know better”. Sara referred to the lack of sexual education when she was young and stressed its importance for disabled people as preventative knowledge: “I did not get any sex education as a teenager. It is important to know what is right and what is wrong”. Some women did not report violence because at the time they did not conceptualize their experiences as criminalized sexual violence and did not know if their experience would fall under existing criminal law. For example, Linda spoke about her lack of knowledge on legislation covering rape and wondered if what she experienced would qualify as such under the law.

In addition to the individual lack of timely knowledge on sexual violence, the women referred to a community of silence and general misunderstanding of sexual relationships and violence. Linda repeated her lack of prior knowledge about rape and sexual violence. As a result, this prevented her from interpreting an assault as an act of violence, and thus, removed the possibility at the time of preventing further sexual abuse. She described one of the instances of how she was subjected to rape by her partner while she was asleep. He told her the next morning what he had done to her and even though she felt violated, she thought this was part of being kinky and in a relationship:

I was violated, deep inside I thought this, but I just thought this was a part of the kinky stuff. I thought: This is allowed when you’re in a relationship. A boyfriend, he cannot rape you.

She recalls discussing these issues with her friends and they concluded “Your boyfriend does not rape you”. A similar lack of timely realization of wrongdoing was also mentioned by Regn, who stated that it took them a long time to realize, address and seek help with dealing with their experiences. For both of these participants, such lack of realization of wrongdoing led them to remain in their respective relationships, and thus, not report their partners. Another aspect of the lack of timely realization and naming the assault as an violence is the case of Lilja, who convinced herself that her friend did not rape her: “I wasn’t really sure, I didn’t tell myself that he had raped me”. She explained that this was due to lack of preventative and timely information and general knowledge on sexual abuse, including rape.

#### Lack of Knowledge on Where to Go for Support or Reporting

For many of the women the lack of information on where to seek support or report violence was also a reason for their inaction in reporting it to the police or Rights Protection Officers, whose role is to assist with the protection of, and enable access to, rights for disabled people. Hanna stated that one of her reasons for not reporting was her lack, at the time, of knowledge of what sexual violence entailed but also not knowing where to go to report it or seek help: “During that time there was no talk about this. I had no idea where to go”. Like Hanna, Runa emphasized her lack of information on how or where to report the sexual harassment she experienced by a male colleague. Even though she told what happened to a female colleague, she was still not informed that she could formally report:

I don't think this matter was taken seriously at all. I was not advised that a complaint could be filed. This has had a great impact on me, and I have lived with it for the rest of my life.

The analysis of the interview data revealed the apparent lack of knowledge regarding support services which, if present, could have connected the women to the reporting structures. Lilja stated that she had no knowledge about what supports existed at the time of the violence. Acknowledging the lack of information on supports, Regn appreciated the understanding and direction of where to go given by a counsellor: “I’m very grateful for that they directed me to Stígamót (centre for survivors of sexual violence) since I didn’t really know much about any of these organizations”. At the time of the interview, some of the women declared not knowing about the existing women's support services or the Right Protection Officers. The participants who knew about such services most often learned about them through engagements with disabled people's organizations. However, for other women who had no connection with disability organizations the knowledge on their part tended to be limited or non-existent. For some, their location outside of the capital region seemed to play a role. For example, as Ella explained: “I don’t have any information or any knowledge about this stuff here in the north”. Others heard of some organizations but did not know much about them. Laura only knew of the Red Cross. Jessie stated regarding Bjarkarhlíð (centre for survivors of violence): “I know that they exist yeah, but I don’t know 100% what they do”. Furthermore, as highlighted elsewhere (Gjecaj et al., 2024) many of the women knew little about the Rights Protection Officers and their role.

#### Interpersonal Barriers

Many of the women described interpersonal-level barriers to reporting. Subcategories include: the relationship to the perpetrator, position of power of the perpetrator, fear of not being believed and lack of trust of authorities, and fear of negative social reactions to disclosure.

#### Relationship With the Perpetrator

Many of the women explained that knowing the perpetrator in some way acted as a barrier to reporting. They defined reporting as difficult depending on who the perpetrators were and in general what they represented to them. Some of the women felt pressure not to report due to allegiances to the perpetrators. This was the case with Linda, who stated that it would have been hard to report her partner due to loving him and the length of their relationship:

He wasn’t all bad… and also what made this so difficult and what I think is also so often forgotten when we talk about violence and pressing charges, is that usually those who violate us, these are the ones that we love the most. I loved this man, even though he was a shit.

This was also the case for Lilja, who explained it would have been hard to report her friend, someone she knew “very well”. Many of the women also listed family members and friends as difficult to report, especially those very close to them

Some women did not report because of feelings of guilt for doing so and the need to protect the perpetrator. For instance, Regn described this guilt as having its origins in a range of factors: that it would not be helpful to them nor their perpetrator to report; a lack of trust of authorities as police might be too hard on the perpetrator; that some of their perpetrators have disabilities such as autism, and with some being children at the time the violence occurred. Similar to Regn, Jessie spoke about guilt and feeling scared of their partner potentially being sent to jail, and worried about how things would be communicated to him by police and other authorities. Lilja explained that she did not report her friend because she did not want to ruin his life: “I know him too well. I don’t want to ruin his life by making it in his permanent record being reported for rape, charged with rape”. For Linda, the feeling of guilt was based on her partner having children and thus not wanting the burden of leaving children without a father:

No, I couldn’t. I tell everybody that asks me (about the violence) but going to the police and stuff like that, I didn’t do that. I could have and I thought about it. (Researcher: was there something that stopped you?) Yes, his boys, definitely that was a part of it, yes, he has children.

#### Fear of Not Being Believed

Perceiving oneself to be in a lower societal status due to age, gender and having a disability, compared to the perpetrator, was voiced as a barrier to reporting. The social influence or status of the perpetrator and consequently the fear of not being believed by justice authorities were cited as major reasons for not reporting. For example, Helga, who did not report the sexual harassment she was subjected to by a doctor, highlighted the power imbalance which led to her fear not being believed:

I didn’t tell anybody about it for at least 10 or 20 years. I thought ‘nobody will believe me’. I thought ‘I should charge him’, but I also thought, ‘I’m a young disabled woman’. I didn’t have much self-confidence at that time and he's a respected doctor. Who will they believe? I was convinced that it was useless for me to do anything because nobody would believe me. The police or judge or whoever would rather believe what he said, than me.

Like Helga, other women also referred to the fear of not being believed by police as a barrier to reporting. Connecting being autistic with lack of trust of authorities, Regn stated that “I have a general distrust of law enforcement. I don’t think they would have our best interest in mind, especially autistic individuals… I wouldn’t be believed”. Laura highlighted language barriers as part of her fear of not being believed as well as her distrust of police, stating that everything is more difficult when you are not from Iceland and speak limited English, which adds more stress and contributes to a general lack of trust.

#### Fear of Negative Social Reactions to Disclosure

Some of the women discussed how the potential of receiving negative public reactions when disclosing their experiences precluded reporting. Potential negative reactions they anticipated included media portrayals, disbelief in their story, shame, embarrassment and fear of retaliation from the perpetrator. In particular, the women highlighted not wanting to report due to fear of negative and hateful social media reactions and other public consequences. For Linda, the fear of how the media would portray her was combined with what might be made public during reporting and the overall justice process. She also feared what her partner would publish on social media. For example, exposing knowledge about “my body and persona … or family” and people's reactions and comments, especially on social media platforms. She considered not reporting as a “kind of protecting myself”. In her words:

I was afraid of what he (the partner) would say, that he would go out and tell something that I didn’t want to be public. So, I was also protecting myself. I know that these courageous women who go and press charges, they are often so harshly critiqued because of shit these guys tell about them, ‘she was like this’ and ‘she was like that’.

Hence, the desire to maintain privacy also kept her from disclosing and reporting.

Connected to the fear of negative media portrayals were potential implications for their public image. Both Linda and Lilja were concerned with their public image and wanted to avoid being primarily known as rape victims. Linda was sure that if she had reported, her life would not be the way it is now: “For me I just couldn’t do it. I know that if I would have done it, my life would not be where it is now. I know it would not. I just can’t be that hero”. Lilja, on the other hand, spoke about the stigma attached if she had reported and the media shadow of rape if she were to become a public figure in the future: “You, being public, your name and face” and “the stigma if you want to become someone in the future”.

#### Cultural and Structural Barriers

Cultural and structural barriers to reporting included societal norms, fears about the difficulty in securing a conviction, inaccessible and costly justice system, and the perception of police.

#### Societal Norms

Some women discussed how societal norms, such as community silence and sexual taboos, precluded reporting. As discussed under personal barriers, the women referred to the existence of a culture of silence about violence in the society and a general misunderstanding of sexual relationships and what constitutes violence within them. This study concerns disabled women's experience of violence in the period between 1980 and 2022. Sadly, their stories reflect that lack of information, community silence and absence of identifying sexual violence, seemed to have prevailed for decades. Helga highlighted that when she was a young disabled woman and subjected to sexual harassment, no one talked about these things. Hanna confirmed this by stating the same: “During that time there was no talk about this”. Similarly, Lilja, a disabled woman in her 20s at time of interview, who was subjected to rape almost 40 years after Helga, also emphasized the lack of information and knowledge in society by stating that “It wasn’t talked about, rape and everything. You didn’t know what was wrong or right”. Many of the women also discussed other societal norms, such as expectations for women and the marginalization and stigmatization of disabled people, which lead to how disabled women are perceived in society and lack of credibility attached to them.

The participants also emphasized the nature of public discourse which tolerates fun made at the expense of disabled women and how matters regarding them are often portrayed in a demeaning manner. Linda stressed the social media effect and how women are “scolded” in comments sections. She emphasized that such public reactions to disabled women was part of her reason for not reporting. Having observed the negative comments when a case involving violence of a disabled woman becomes public, Lilja stated that if she was to report she would not want it to be noted that she is disabled. She highlighted the stigma attached to this identity and the othering of disabled people within the media and community. Participants also highlighted that given Iceland's relatively small population and its closely interconnecting social networks, it is much more difficult to hold a person accountable for violence against disabled women if they were well-connected socially.

#### Inaccessible and Expensive Justice System

Some women expressed personal dissatisfaction and mistrust of the criminal justice system due to having watched how many perpetrators walk free. They also highlighted the cost of the justice system and lack of legislative protections as barriers. Expressing a sense of hopelessness, Lilja noted her reason for not reporting as the difficulties of going through the justice system: “Because I’m not positive that he will be charged, that it will go through because it's so rare for cases to go to court and unlikely for me to win the case”. Sandra highlighted that the justice system is expensive. She pointed out that the fear of losing and having to pay a lot of money are among the reasons for not reporting violence. She added that financially there is no equal access to justice, especially for people in her positions.

#### Perception of Police

Another aspect that acts as a barrier to reporting regards immigrant women in particular and their perceptions of police in their country of origins which influenced whether they would report violence to police in Iceland. This was the case for both of the foreign participants. Jessie had reservations about contacting the police and thus did not report the domestic violence to police nor to the Right Protection Officers. Trying to explain their reluctance, they referred to their upbringing and portraits of police on TV:

Police, mostly police men were presented like the idiot who wants to put everyone in jail just to get his quota. So, actually that's where I started having huge panic attacks even as a four-year-old when I saw policemen, or even people with similar hats like train officers. So, this is not a good basis… to go to the police when there is a problem.

In addition, Jessie described an incident at an airport in their country of origin where the police were rude, angry, and insisted on proof of their disability because they had gone to the disability assistance line. These experiences seemed to influence if they would contact the police even if it was in a different country, despite the fact that they stated they were not scared of police in Iceland. Like Jessie, Laura was also hesitant if she would report given her experience with police in her country of origin:

I don’t know, … maybe I’m scared to go to the police because I’m scared of everything, also of police because I have a posttraumatic syndrome after (country of origin). I am scared here in taxi also because here taxi is like big black car, in (country of origin) are police cars.

In comparison to the two foreign women, Iva, an Icelandic woman who was studying abroad, stated that she would not have sought support or report violence there. She believes that because she is a foreigner she would be treated differently to natives.

### Reporting the Violence

Following our analysis of the multiple and complex barriers to reporting, this section discusses the experiences of the three women who did report the violence they experienced. Their reasons for reporting varied and so did the outcomes of doing so. For example, Mary, a young woman, who was sexually assaulted by a staff member where she received services, first disclosed her experience to her mother. Her mother believed her without question and this support reduced Mary's fears that she would not be believed. This made it easier for her to tell her story to others and to report the assault to the police. Mary also received support from a Right Protection Officer, who facilitated access to justice by providing procedural accommodation and which made her feel “safer and comfortable” while giving her victim/witness statement to the police and in court. This support was undoubtedly significant in fostering Mary's positive experience of the justice system and confidence in winning the case. In fact, Mary is the only woman in this study whose case went to court and which resulted in a positive outcome. For a detailed description of Mary's court see Gjecaj et al. (2023).

The two other participants who reported, Sandra and Jessie, had been subjected to multiple forms of violence. Sandra reported digital hate speech to the police at different times in her life, which was perpetrated by male strangers and other members of society. In her words “I experienced online abuse quite a lot … that went completely crazy … and I started to get personal letters and phone calls with threatening stuff”. This left her with no other choice but to call the police. When reporting, she appreciated the validation that she received from the police of her fear, which made her feel believed and relieved that she did not have to convince the police officer of the violence she had experienced. Understanding that there was nothing more she could do due to lack of legislation pertaining to online abuse at the time, she felt that the action taken by police was enough: “He said that he will have someone nearby the area over the weekend. So, I had the police cruising around for few days … and I felt, that was enough for me at that time”.

The third participant to report was Jessie. They were subjected to domestic violence by their male partner. They had reservations about contacting the police due to their perceptions of police in their country of origin. Instead, they opted to seek help from a Rights Protection Officer who assisted them in addressing issues related to the violence, including assisting Jessie with finding a safe place to live.

### Reporting and Justice

The women in the study had a range of different views and understandings of justice. For those who reported the violence, reporting was part of the process of seeking justice. For example, highlighting that justice is an individual matter, Sandra stated that “I think justice can look differently for each disabled woman, and I think it is a really personal thing”. She emphasized the importance of seeking justice by stating: “Justice is a source of healing; justice is important for the healing process”. Identifying the police action the first time she reported as a basic level of justice, she highlighted that “feeling assured” and to feel that somebody cares is important for the healing process. She also noted that sometimes getting the woman's story heard is a form of justice as well. For Mary, justice was the fact that she was considered to be a credible witness and provided with procedural and reasonable accommodation throughout the process. The case ended up in court and was successful, the perpetrator was convicted, and she was awarded some remuneration. Jessie did not specifically refer to justice. However, while appreciating the support provided by the Rights Protection Officer, they stated that their reporting of the violence resulted in an outcome that they found just and helpful.

The women who did not report expressed a belief that reporting would not result in justice. Each of the women described their individual views on justice: what they would have liked to have happen and what they saw as justice. They included factors such as distancing from the perpetrators, admissibility and accountability, and non-discriminatory services as justice instead of reporting. Repeating their lack of trust in the police, Regn stated that reporting would not have been of any help and was concerned that it could instead have potentially put them at risk of further violence committed by their original perpetrators:

I just don’t think that it would actually help me to report it because I’ve already just distanced myself from the individuals at fault and I can’t really think of anything that reporting would actually improve. I think it would, in at least some cases, be actually detrimental to the actual abusers. I think they wouldn’t understand what's happening or somehow get tipped off that it was me who was at fault and maybe sort of rekindle their rage which would just put me at risk again.

Regn believed that reporting would not bring them justice. On the contrary, they believed it would bring about negative and potentially dangerous consequences. Distancing themselves from the perpetrators to avoid further abuse was, in their view, the best outcome. Similar to Regn, Lilja also referred to reporting as not resulting in justice for her. After a few years, the friend who raped her texted to apologize without referring to what he was apologizing for. Even though it was a vague text, she felt relieved, and it enabled her to have some kind of closure. She repeated that his admission was her justice: “Because it is enough for me just that he's admitting what he did was wrong, … yes, I don’t need the justice on paper, on records, that's not what I need”. Referring to system's violence, Kolbrún explained that to her, justice means not constantly having to apply and fight for services, and not be discriminated against because her disability is not visible.

For many of the women, instead of reporting their perpetrators, education, finding employment, receiving professional help, living free from violence and becoming an advocate served as forms of justice. Linda explained that justice was getting out of the abusive relationship: “Yes, that's justice for me and I also wanted just to put this behind me, I wanted this to be finished. Never again”. For Helga justice came in the form of receiving professional assistance enabling her to become an advocate raising awareness about violence and telling other disabled women about her experiences.

## Conclusion

This paper has highlighted the significant and multiple barriers to reporting violence encountered by the vast majority of the disabled women in the study. We have broadly categorized the barriers described by the women as *personal*, *interpersonal*, and *cultural and structural*. While the existing international literature discussed in the introduction does mention similar barriers as those identified in this study, they lack a comprehensive overview. Addressing this gap and aiming to provide an in-depth understanding, this paper has specifically focused on the barriers to reporting violence based on the voices and lived experiences of the disabled women who took part in this study. These barriers show that education and awareness raising regarding violence and access to justice are desperately needed. Our findings also highlight the lack of information and availability of a range of support services for disabled women that can assist them in identifying violence and abuse, facilitate their process through the justice system and make sure they receive professional support services to process the effects of being violated.

We recommend that provision of sexual education and awareness raising about violence among disabled people and the general public as a preventive and protecting measure. Efforts should be made to enable reporting through increased education and training within the justice system and strengthening of procedural and reasonable accommodation. We also call for the availability of qualified support for disabled victims and perpetrators, including in particular Rights Protection Officers. Addressing the fear of not being believed, listened to, or fear of being subjected to further victimization by the police is a necessary step to enable reporting of violence by disabled women. Making sure more cases be prosecuted and processed through the justice system would also be an important step to show dedication to addressing the disabled women's concerns and signal to them, and the general public, that such violence is criminalized and not tolerated.

Overall, we contend that reporting is a consideration which varies from person to person. Support and accommodation must be ensured for those who wish to report as well as making available various forms of support for those who do not wish to do so. Part of such accommodation is making sure that information about legal rights as well as new developments reach disabled women. Reporting is not always the sought after form of justice, nor sometimes even a part of it. Justice is an individual pursuit and can take on different shapes depending on what each person considers as justice. However, living free from violence and accessing justice is a human right, and as such these varied paths to justice for disabled women must be respected, institutionalized and supported.
